# Supplementary material for: Evolution of sensory systems underlies the emergence of predatory feeding behaviors in nematodes
Source: Proc Natl Acad Sci U S A. 2026 Jan 28;123(5):e2514172123. doi: 10.1073/pnas.2514172123 (PMC12867699; doi:10.1073/pnas.2514172123)
Supplement: Supplementary file 1 — Appendix 01 (PDF) [file pnas.2514172123.sapp.pdf]

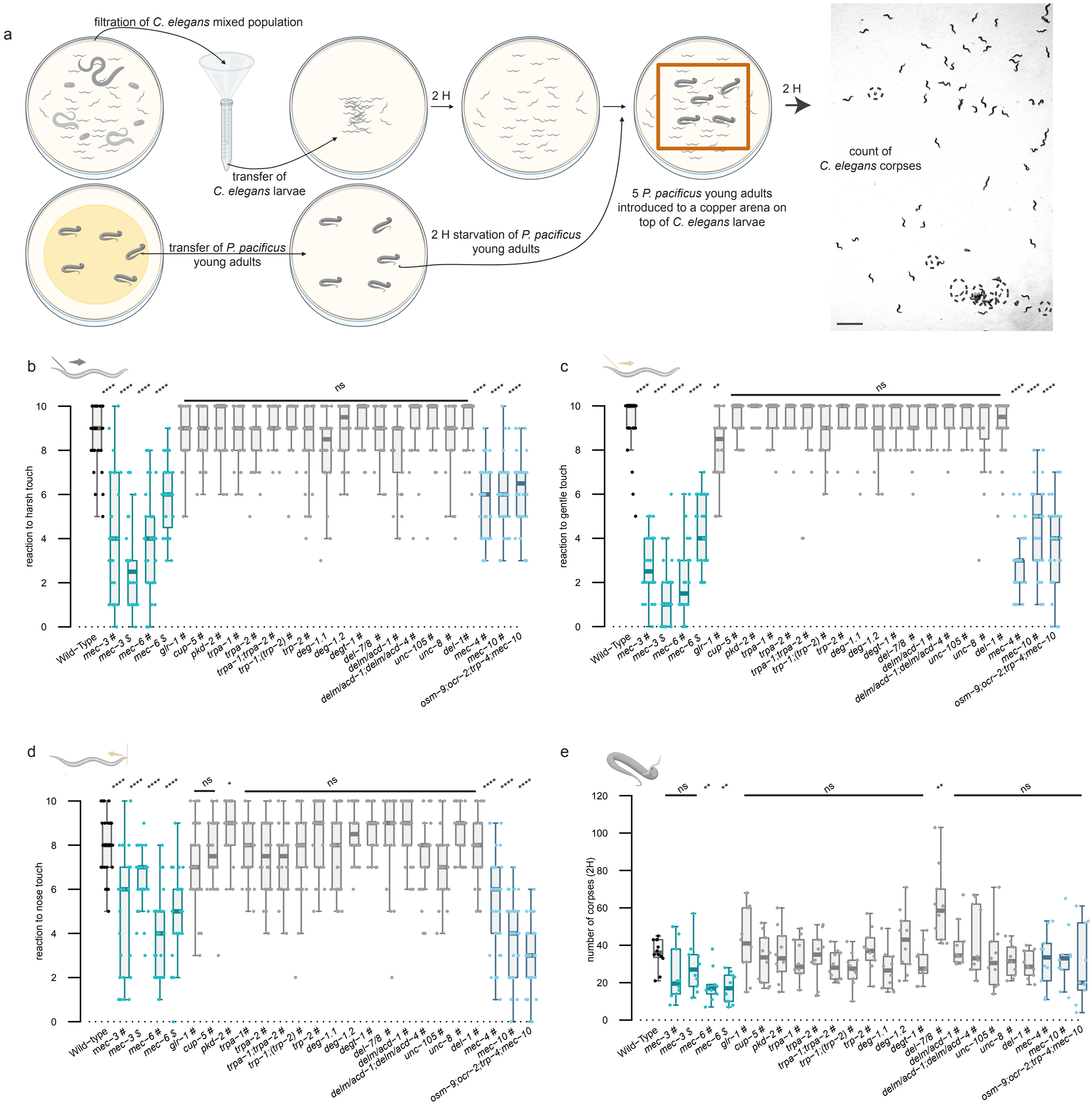

a
filtration of C. elegans mixed population
transfer of
C. elegans larvae
2 H
transfer of P. pacificus
young adults
2 H starvation of P. pacificus
young adults
5 P. pacificus young adults
introduced to a copper arena on
top of C. elegans larvae
2 H
count of
C. elegans corpses
b
reaction to harsh touch
c
reaction to gentle touch
d
reaction to nose touch
e
number of corpses (2H)
ns
Wild-Type
mec-3 #
mec-3 $
mec-6 #
mec-6 $
glr-1 #
cup-5 #
pkd-2 #
trpa-1 #
trpa-2 #
trpa-1;trpa-2 #
trp-1;(trp-2) #
trp-2 #
deg-1.1
deg-1.2
degt-1 #
del-7/8 #
delm/acd-1 #
delm/acd-1;delm/acd-4 #
unc-105 #
unc-8 #
del-1 #
mec-4 #
mec-10 #
osm-9;ocr-2;trp-4;mec-10

Supplementary Figure 1: Mechanosensation and predation in *P. pacificus*

(A) Schematic of the corpse assay protocol. Five starved *P. pacificus* predators of the strain of interest are introduce in an area with an abundancy of *C. elegans* larvae. After 2 h, predatory success is assessed by counting the number of larval corpses inside the arena. In the image corpses can be observed inside the circles. Scale bar is 500 µm. (B) Mechanosensory assays to harsh touch, (C) gentle touch, and (D) nose touch. Each assessment is the result of ten consecutive trials of each worm. At least 30 worms were tested per strain. (E) Number of *C. elegans* corpses counted after two hours of contact with the indicated *P. pacificus* strains as predator. At least 10 assays were performed. Statistical tests: two-tailed Wilcoxon Mann Whitney with Benjamini-Hochberg correction, non-significant (ns), p-value ≤ 0.05 (*), ≤ 0.01 (**), ≤ 0.001 (***), ≤ 0.0001 (****). Schematics were made with biorender.

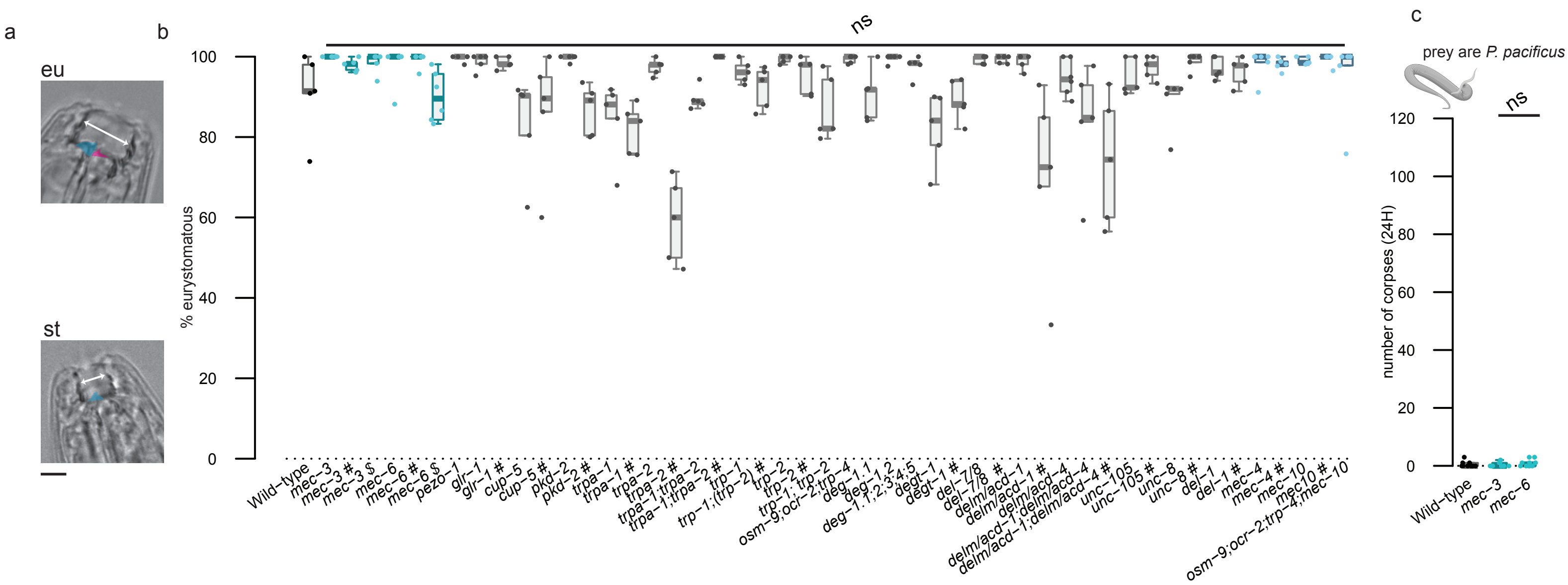
a
eu
st
b
ns
% eurystomatous
100
80
60
40
20
0
Wild−type
mec−3
mec−3 #
mec−3 $
mec−6
mec−6 #
mec−6 $
pezo−1
glr−1
glr−1 #
cup−5
cup−5 #
pkd−2
pkd−2 #
trpa−1
trpa−1 #
trpa−2
trpa−2 #
trpa−1;trpa−2
trpa−1;trpa−2 #
trp−1
trp−1;(trp−2) #
trp−2
trp−2 #
trp−1; trp−2
osm−9;ocr−2;trp−4
deg−1.1
deg−1.2
deg−1.1;2;3;4;5
degt−1
degt−1 #
del−7/8
del−7/8 #
delm/acd−1
delm/acd−1 #
delm/acd−4
delm/acd−4
delm/acd−1;delm/acd−4
delm/acd−1;delm/acd−4 #
unc−105
unc−105 #
unc−8
unc−8 #
del−1
del−1 #
mec−4
mec−4 #
mec−10
mec10 #
osm−9;ocr−2;trp−4;mec−10
c
prey are P. pacificus
ns
number of corpses (24H)
120
100
80
60
40
20
0
Wild−type
mec−3
mec−6

Supplementary Figure 2: Mechanosensation does not affect developmental plasticity or kin-recognition

(A) *P. pacificus* is developmentally plastic leading to one of two mouth forms, stenotomatous mouth (st, bottom) with the dorsal tooth (blue) or eurytomatous (eu, top) with an additional subventral tooth (red) and a wider opening. Scale bar is 10 µm. (B) Percentage of eu was assess 5 time for each strain. (C) Number of wild-type *P. pacificus* corpses counted after 24 hours of contact with the indicated *P. pacificus* strains as predator. Statistical tests: two-tailed Wilcoxon Mann Whitney with Benjamini-Hochberg correction, non-significant (ns), p-value ≤ 0.05 (*), ≤ 0.01 (**), ≤ 0.001 (***), ≤ 0.0001 (****). Schematics were made with biorender.

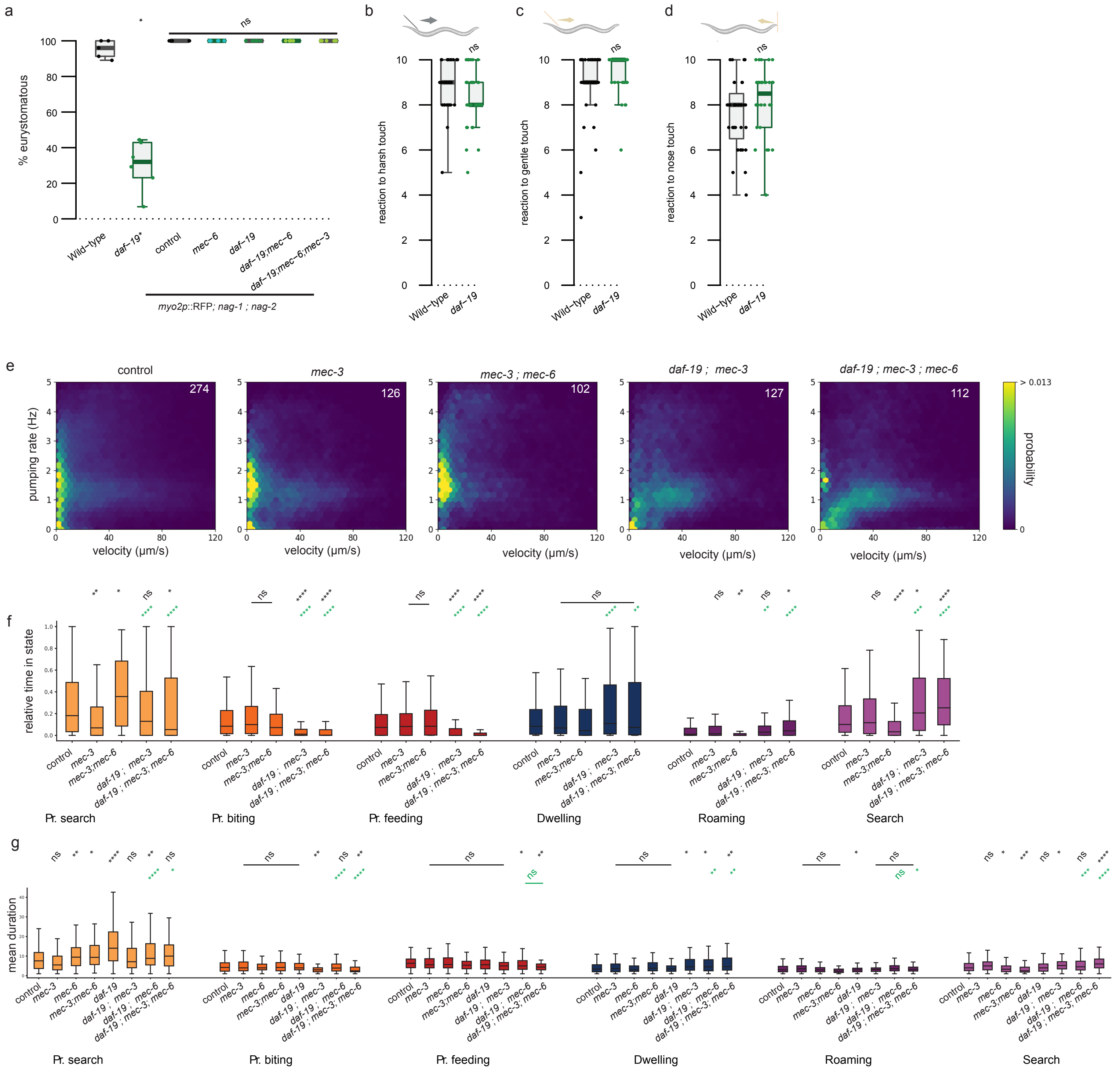
a
% eurystomatous
Wild-type
daf-19*
control
mec-6
daf-19
daf-19;mec-6
daf-19;mec-6;mec-3
myo2p::RFP; nag-1 ; nag-2
ns
b
reaction to harsh touch
c
reaction to gentle touch
d
reaction to nose touch
Wild-type
daf-19
e
control
mec-3
mec-3 ; mec-6
daf-19 ; mec-3
daf-19 ; mec-3 ; mec-6
274
126
102
127
112
pumping rate (Hz)
velocity (µm/s)
probability
> 0.013
f
relative time in state
Pr. search
Pr. biting
Pr. feeding
Dwelling
Roaming
Search
g
mean duration
Pr. search
Pr. biting
Pr. feeding
Dwelling
Roaming
Search

Supplementary Figure 3: Behavioural analysis of sensory deficient worms

(A) Percentage of eu morphs were assessed 5 time for each strain. As shown in Moreno et al 2019 (1) , mutations in *Ppa-daf-19* lead to a higher occurrence of st mouth form which is overcome by including mutations in *Ppa-nag-1* and *Ppa-nag-2*. *daf-19** is the only time mutation of *Ppa-daf*-19 is not studied in a strain also mutated for *Ppa-nag-1* and *Ppa-nag-2.* (B) Mechanosensory assays to harsh touch, (C) gentle touch, and (D) nose touch. Each assessment is the result of ten consecutive trials of each worm. At least 30 worms were tested per strain. (E) Joint probability density map of velocity (μm/s) and pumping rate (Hz) for animals corresponding to the indicated genotypes. The number of worms is indicated in the top right corner. (F) Time spent in each behavioural state normalized to the total track duration. (G) Mean duration of each behaviour in B. Statistical tests: Significance from comparison to wild-type (black) and to *daf-19* single mutant (green) was assessed using two-tailed Wilcoxon Mann Whitney with Benjamini-Hochberg correction(A-D), Mann-Whitney U-test with a Bonferroni correction (F-G). non-significant (ns), p-value ≤ 0.05 (*), ≤ 0.01 (**), ≤ 0.001 (***), ≤ 0.0001 (****). Schematics were made with biorender. Predatory (Pr.)

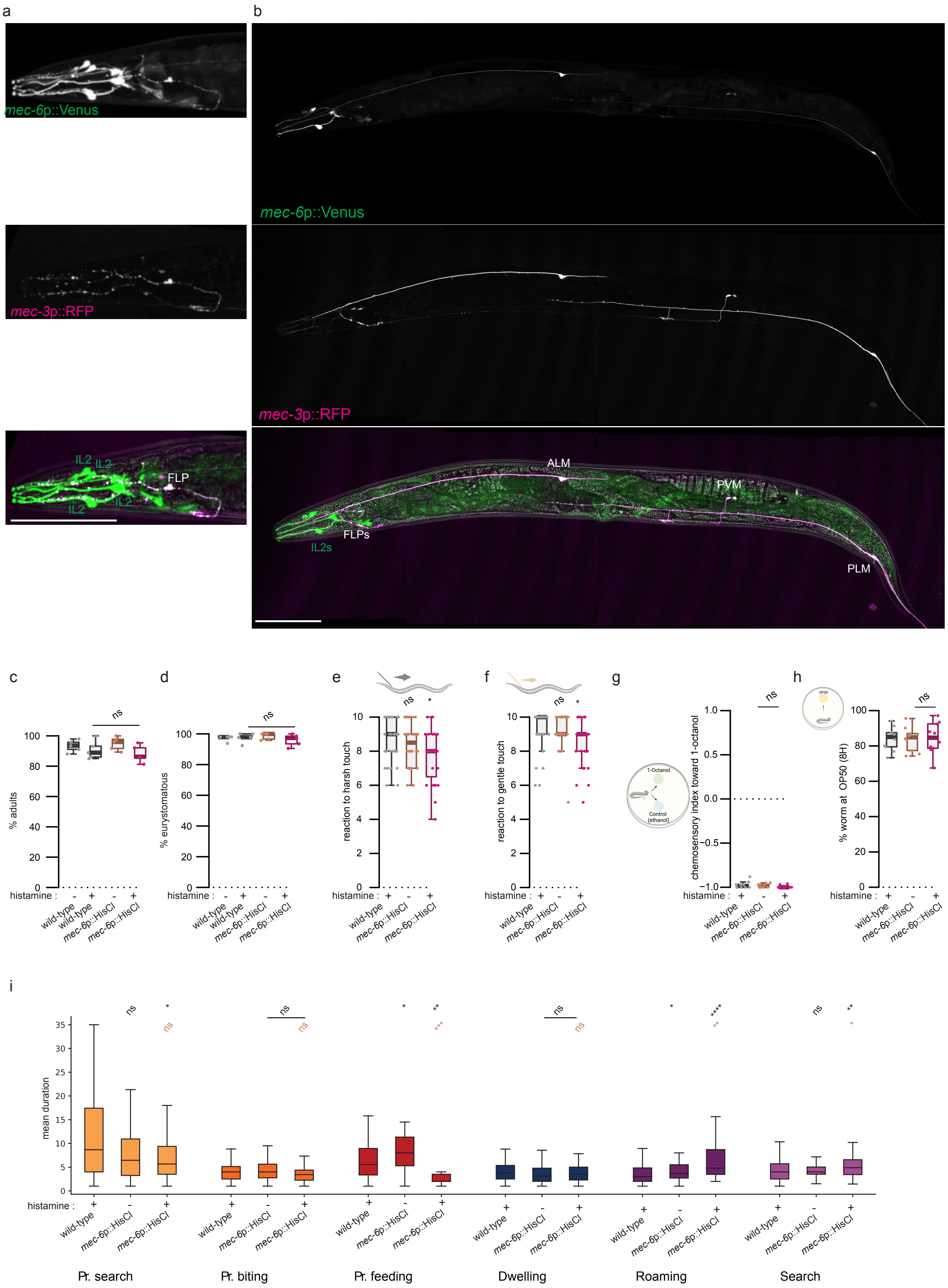
a
b
mec-6p::Venus
mec-3p::RFP
IL2
FLP
ALM
PVM
FLPs
IL2s
PLM
c
d
e
f
g
h
i
% adults
% eurystomatous
reaction to harsh touch
reaction to gentle touch
chemosensory index toward 1-octanol
1-Octanol
Control (ethanol)
OP50
% worm at OP50 (8H)
ns
histamine :
wild-type
mec-6p::HisCl
mean duration
Pr. search
Pr. biting
Pr. feeding
Dwelling
Roaming
Search

Supplementary Figure 4: touch neurons in *P. pacificus*

(A) Head of a worm expressing *mec-6p*::Venus (top, green) and *mec-3p*::RFP (middle, magenta). Co-expression in the FLP of both *Ppa-mec-6* and *Ppa-mec-3* is visible in the merge image (bottom). IL2 neurons are indicated. Scale bar is 100 µm. (B) Representative image of a worms expressing *Ppa-mec-6p*::Venus (top, green) and *Ppa-mec-3p*:: RFP (middle, magenta) and the merge (bottom). Putative *P. pacificus* neuronal identity, based on *C. elegans* soma placement and known mechanosensory function, is indicated in white for co-expression. Scale bar is 100 µm. (C) Percentage of worms reaching adulthood after 4 days was assessed 5 time for each condition: wild-type worms and worms expressing histamine chloride channel under the control of *Ppa-mec-6* promotor in absence (-) or presence (+) of histamine. (D) Percentage of eu mouth form animals was assessed 5 time for each condition. (E) Mechanosensory assays to harsh touch and (F) gentle touch for worms of each condition. Each data point is the result of ten consecutive trials for a single worm. At least 30 worms were tested per condition. (G) Chemotaxis index analysing the aversive response to 1-octanol. Each condition was tested 10 time. (H) Percentage of worms finding a bacterial OP50 food source after 8 hours. Each condition was tested 10 time. (I) Mean duration of each behavioural state. Statistical tests: Significance from comparison to wild-type (black) and to *Ppa-mec-6p*::HisCl untreated worms (brown) was assessed using two-tailed Wilcoxon Mann Whitney with Benjamini-Hochberg correction (C-H), Mann-Whitney U-test with a Bonferroni correction (I). non-significant (ns), p-value ≤ 0.05 (*), ≤ 0.01 (**), ≤ 0.001 (***), ≤ 0.0001 (****). Predatory (Pr.) Schematic was made with biorender.

Table S1. List of all strains used throughout study.

Name in article	strain name	strain background	genotype	source
Wild-type	PS312	PS312	/	Sommer lab
mec-3	JWL74	PS312	*mec-3(bnn60)*	this study
mec-3 #	JWL75	PS312	*mec-3 (bnn61)*	this study
mec-3 $	JWL110	PS312	*mec-3 (bnn94)*	this study
mec-6	JWL79	PS312	*mec-6 (bnn67)*	this study
mec-6 #	JWL84	PS312	*mec-6 (bnn70)*	this study
mec-6 $	JWL78	PS312	*mec-6 (bnn66)*	this study
pezo-1	JWL26	PS312	*pezo-1 (bnn20)*	this study
glr-1	JWL46	PS312	*glr-1 (bnn34)*	this study
glr-1 #	JWL43	PS312	*glr-1 (bnn31)*	this study
cup-5	JWL102	PS312	*cup-5(bnn90)*	this study
cup-5#	JWL101	PS312	*cup-5(bnn89)*	this study
pkd-2	JWL100	PS312	*pkd-2 (bnn88)*	this study
pkd-2#	JWL108	PS312	*pkd-2 (bnn93)*	this study
trpa-1	JWL82	PS312	*trpa-1 (bnn68)*	this study
trpa-1#	JWL81	PS312	*trpa-1 (bnn62)*	this study
trpa-2	JWL94	PS312	*trpa-2 (bnn82)*	this study
trpa-2#	JWL85	PS312	*trpa-2 (bnn63)*	this study
trpa-1; trpa-2	JWL76	PS312	*trpa-1 (bnn62) ;trpa-2 (bnn63)*	this study
trpa-1;trpa-2#	JWL95	PS312	*trpa1(bnn83); trpa-2(bnn84)*	this study
trp-1	JWL72	PS312	*trp-1 (bnn59)*	this study
trp-1;(trp-2)#	JWL65	PS312	*trp-1(bnn50); trp-2(bnn51)*	this study
trp-2	JWL67	PS312	*trp-2 (bnn54)*	this study

trp-2#	JWL71	PS312	*trp-2 (bnn58)*	this study
trp-1; trp-2	JWL66	PS312	*trp-1(bnn52); trp-2(bnn53)*	this study
osm-9;ocr-2;trp-4;	JWL16	PS312	*trp-4 (bnn11); ocr-2(bnn8); osm-9(bnn9)*	this study
deg-1.1	JWL88	PS312	*deg-1.1 (bnn73)*	this study
deg-1.2	JWL87	PS312	*deg-1.2 (bnn72)*	this study
deg-1.1;2;3;4;5	JWL89	PS312	*deg-1.1(bnn76);deg-1.2(bnn72);deg-1.3(bnn74);deg-1.4(bnn75);deg-1.5(bnn77)*	this study
degt-1	JWL86	PS312	*degt-1 (bnn71)*	this study
degt-1#	JWL83	PS312	*degt-1 (bnn69)*	this study
del-7/8	JWL41	PS312	*del-7/8 (bnn29)*	this study
del-7/8#	JWL42	PS312	*del-7/8 (bnn30)*	this study
delm/acd-1	JWL91	PS312	*delm/acd-1 (bnn79)*	this study
delm/acd-1#	JWL92	PS312	*delm/acd-1 (bnn80)*	this study
delm/acd-4	JWL80	PS312	*delm/acd-4(bnn65)*	this study
delm/acd-1;delm/acd-4	JWL93	PS312	*delm/acd-1 (bnn80) ;delm/acd-4(bnn81)*	this study
delm/acd-1;delm/acd-4#	JWL77	PS312	*delm/acd-1 (bnn64); delm/acd-4(bnn65)*	this study
unc-105	JWL97	PS312	*unc-105(bnn86)*	this study
unc-105#	JWL99	PS312	*unc-105(bnn87)*	this study
unc-8	JWL96	PS312	*unc-8(bnn85)*	this study
unc-8#	JWL103	PS312	*unc-8 (bnn91)*	this study
del-1	JWL199	PS312	*del-1 (bnn157)*	this study
del-1#	JWL200	PS312	*del-1 (bnn158)*	this study
mec-4	JWL63	PS312	*mec-4 (bnn49)*	this study
mec-4#	JWL62	PS312	*mec-4 (bnn48)*	this study
mec-10	JWL22	PS312	*mec-10 (bnn16)*	this study
mec10#	JWL20	PS312	*mec-10 (bnn14)*	this study
osm-9;ocr-2;trp-4;mec-10	JWL24	PS312	*mec-10 (bnn18); trp-4 (bnn11); ocr-2(bnn8); osm-9(bnn9)*	this study

*daf-19**	RS3238	PS312	*daf-19 (tu1167)*	Moreno et al. 2019 (1)
control	JWL147	PS312	*bnnls1 [myo-2p::RFP;egl20p::GFP] ; nag 1 (bnn100) , nag-2 (bnn101)*	this study
mec-3	JWL157	PS312	*bnnls1 [myo-2p::RFP;egl20p::GFP] ; nag 1 (bnn100) , nag-2 (bnn101);mec-3(bnn60)*	this study
mec-6	JWL176	PS312	*bnnls1 [myo-2p::RFP;egl20p::GFP] ; nag 1 (bnn100) , nag-2 (bnn101) ; mec-6(bnn66)*	this study
mec-3; mec-6	JWL158	PS312	*bnnls1 [myo-2p::RFP;egl20p::GFP] ; nag 1 (bnn100) , nag-2 (bnn101); mec-6(bnn66);mec-3(bnn60)*	this study
daf-19	JWL118	PS312	*bnnls1 [myo-2p::RFP;egl20p::GFP] ; nag 1 (bnn100) , nag-2 (bnn101); daf-19(tu1167)*	this study
daf-19; mec-3	JWL159	PS312	*bnnls1 [myo-2p::RFP;egl20p::GFP] ; nag 1 (bnn100) , nag-2 (bnn101); daf-19(tu1167); mec-3(bnn60)*	this study
daf-19; mec-6	JWL119	PS312	*bnnls1 [myo-2p::RFP;egl20p::GFP] ; nag 1 (bnn100) , nag-2 (bnn101); daf-19(tu1167); mec-6(bnn66)*	this study
daf-19; mec-6; mec-3	JWL156	PS312	*bnnls1 [myo-2p::RFP;egl20p::GFP] ; nag 1 (bnn100) , nag-2 (bnn101); daf-19(tu1167); mec-6(bnn66);mec-3(bnn60)*	this study
mec-6p::Venus;mec-3p::RFP	JWL262	PS312	*mec-6p::Venus;mec-3p::RFP [bnnEX23]*	this study
mec-6p::Venus;daf-19p::RFP	JWL201	PS312	*mec-6p::Venus;daf-19p::RFP [bnnEX12]*	this study
mec-6p::HisCl	JWL237	PS312	*mec-6p::Venus;mec-6p::HCl [bnnEX17]*	this study
control	JWL27	PS312	*bnnls1 [myo-2p::RFP;egl20p::GFP]*	Eren et al. 2024 (2)
mec-6p::HisCl	JWL248	PS312	*bnnls1 [myo-2p::RFP;egl20p::GFP] ; mec-6p::Venus;mec-6p::HCl [bnnEX17]*	this study

SI References

1. E. Moreno, J. W. Lightfoot, M. Lenuzzi, R. J. Sommer, Cilia drive developmental plasticity and are essential for efficient prey detection in predatory nematodes. *Proc Royal Soc B* **286**, 20191089 (2019).

2. G. G. Eren, *et al.*, Predatory aggression evolved through adaptations to noradrenergic circuits. *bioRxiv* 2024.08.02.606321 (2024). https://doi.org/10.1101/2024.08.02.606321.
